# Substance Addiction in Adolescents: Influence of Parenting and Personality Traits

**DOI:** 10.3390/brainsci14050449

**Published:** 2024-04-30

**Authors:** Irene Escamilla, Nerea Juan, Ana Benito, Francisca Castellano-García, Francesc Rodríguez-Ruiz, Gonzalo Haro

**Affiliations:** 1 Department of Mental Health, Consorci Hospitalari Provincial de Castelló, 12002 Castelló de la Plana, Spain; 2 TXP Research Group, Universidad Cardenal Herrera-CEU, CEU Universities, 12006 Castelló de la Plana, Spain; 3 Torrente Mental Health Unit, Hospital General Universitario de Valencia, 46014 Valencia, Spain; 4 Department of Educational Sciences, Universidad Cardenal Herrera-CEU, CEU Universities, 12006 Castelló de la Plana, Spain

**Keywords:** parental socialization, personality traits, substance use, psychopathology, adolescence

## Abstract

Background: Substance use in adolescents has been separately related to personality traits and parental socialization styles; in this study, our objective was to study these variables in an integrated way. Methods: A cross-sectional observational study was conducted in five institutes in a final sample of 331 students, excluding those with gaming disorder. The sample was stratified into three subgroups: ‘no addiction’, ‘low risk’, and ‘high risk’ of Substance Use Disorders (SUD). Results: 12.9% of the adolescents presented a low risk of SUD, while 18.3% showed a high risk, with both being older (F = 9.16; *p* < 0.001) than the no addiction group. Adolescents with high risk scored lower in control and structure variables and higher in maternal and paternal indifference factors. Non-addicted subjects presented higher scores in conscientiousness, extraversion, and agreeableness and lower scores in neuroticism. The probability of SUD increased with age (OR = 2.187; *p* = 0.022), sensation seeking (OR = 1.084; *p* < 0.001), and neuroticism (OR = 1.049; *p* = 0.042), while conscientiousness was a protective factor (OR = 0.930; *p* = 0.008). Conclusions: These results reflect that personality traits are directly related to the development of substance abuse in adolescents.

## 1. Introduction

Adolescence is a stage of life in which several changes occur at a cognitive, emotional, physical, and social level, being particularly sensitive to social and environmental models, which makes it a critical period for the emergence of both risky and addictive behaviors [1]. Some studies suggest that more than 80% of adolescents experiment with drugs or alcohol before adulthood [2].

Current biological models of adolescent vulnerability to addictions incorporate changes in the function and structure of the midbrain dopaminergic system, stress-associated neuroplasticity, and maturational imbalances between cognitive control and reward reactivity. A model that explains adolescent addiction and risky behavior involves the interface of 3 neurobiological systems: a control/regulatory system involving the medial and ventral Prefrontal Cortex, a reward (approach) system involving the ventral striatum and midbrain dopaminergic system, and a threat (harm-avoidance) system involving the amygdala. In this model, an inefficient regulatory system, a strong reward system, and a weak harm-avoidance system contribute to increased engagement in substance use and risky behaviors. Adolescents are more likely than adults to choose smaller immediate rewards over larger delayed rewards, which seems to be associated with activity in the ventral striatum and ventromedial prefrontal cortex (areas that are among the last brain regions to reach maturation, and this may contribute to the specific vulnerability of adolescents to addictions and risk behaviors) [3].

There are many types of family, and the definition of the term is changing according to the places, times, and conditions in which they are located. Family is the first socializing agent children experience. This represents the most significant agent in children’s lives, as through it, they develop essential skills and abilities that facilitate their integration and adaptation in the world [4]. It is considered one of the most relevant elements within the sociocultural factor of the child, as it exposes models of behavior, discipline, attitudes, etc. It thus becomes an influential factor in their psychosocial development [5].

Upbringing constitutes the environment in which values, beliefs, norms, and habits are acquired that will allow children to interact with the world. These educational parenting processes are transmitted through the so-called parenting styles present in every family, through which parents interact with their children [6].

In Baumrind’s studies [7,8,9,10], two underlying dimensions of parent-child relationships were identified: acceptance (the extent to which parents are receptive and loving toward their child) and parental control (the extent to which parents expect mature behavior and exercise control over their child) [11]. These two central dimensions of parenting represent two patterns of parental behavior that, when combined, produce four styles of parental socialization: authoritative, when clear limits are established while parents are also sensitive to their child’s needs; negligent, when there is a lack of involvement in raising the child, without providing the necessary support; authoritarian, when parents show control and severe discipline without considering the needs of the child; and permissive, when they show excessive tolerance towards the child, without imposing limits [12].

The quality of relationships between parents and children considerably influences many decisions and behaviors during early adolescence, with substance use particularly standing out. Educational styles have been studied and have been identified as one of the most notable risk and protective factors against this problem during adolescence. Therefore, parental styles and other factors will influence whether adolescents have a high risk of SUD (i.e., a strong likelihood of developing SUD), a low risk, or no significant risk of addiction [13].

In the literature published to date on the relationship between parental socialization style and substance use in adolescence, the data show a greater risk of abuse among minors who received negligent educational styles when they had felt neglected by their parents [14,15,16,17], or to a lesser extent, those who had experienced authoritarian educational styles. In turn, the authoritative educational style has been associated with a lower risk of substance use disorder [12,18,19,20]. Thus, it has been proposed that the authoritative parenting style can be considered more protective and beneficial to the proper development of young people because this style helps achieve positive bio-psycho-social-spiritual adjustment and greater academic success and, therefore, is more likely to prevent the use of substances and related problems [21].

Moreover, personality traits that favor consumption have also been described, with high scores in neuroticism and impulsivity and low scores in extroversion and sociability among adolescent consumers, especially standing out [22,23]. A relationship between both personality [24] and parental socialization [25] with gaming disorder has also recently been demonstrated, with several studies finding high comorbidity between substance use and internet gaming disorder [26], as well as studies that identify risk factors for comorbidity between gaming disorder and psychopathology (denominated dual disorder) [27]. However, behavioral addictions such as gaming disorders have not been considered in most studies examining adolescents, which may have biased their results. Taking into account that adolescence is a period of special vulnerability for the development of addictions due to neurobiological reasons besides personality is not only genetically predetermined but also influenced by socioenvironmental factors, with educational style being one of the most determining factors in this sense [23], this study aimed to (1) evaluate which parental socialization styles influence the development of substance addiction in the adolescent population and (2) determine the influence of personality traits in SUD while trying to avoid possible biases by excluding adolescents presenting gaming disorder.

## 2. Materials and Methods

### 2.1. Participants

This was an observational and cross-sectional study. The sample comprised 397 students (and their primary caregivers) in the third or fourth years of Compulsory Secondary Education (CSE). They were all from five private subsidized schools and one public school in the province of Castellón (Spain), which were selected by purposive sampling according to availability and geographical location. With the G*Power 3.1.9.4 program, it was calculated that the sample needed to perform ANOVA with three groups (high risk of SUD, low risk of SUD, and no addiction), with effect size 0.25, alpha 95%, and power 80% was 159 subjects. The G*Power software calculates sample size and power for various statistical methods (F, t, χ^2^, Z, and exact tests) [28]. Using the EPIDAT program Version 3.1. [29], we calculated that a sample of 153 participants would be needed to detect a 0.200 correlation between the studied variables with a 95% confidence level and 80% power, and therefore, we confirmed that our sample size was sufficient.

The inclusion criteria were: (1) being in the third or fourth year of CSE and (2) that the adolescent and their main caregiver agreed to participate in the study by signing the informed consent. The exclusion criterion was having some disorder/illness or language difficulties that prevented the completion of the psychometric tests.

### 2.2. Measures

The Alcohol Use Disorders Identification Test (AUDIT) was used to assess excessive alcohol consumption; it contains 10 questions, with the cut-off point being ≥6 in women and ≥8 in men [30]. The AUDIT presents an internal consistency of 0.80 [31], sensitivity of 57–59%, and specificity of 91–96% [32].

The CRAFFT Substance Abuse Screening Instrument (CRAFFT) used to screen for risky alcohol consumption and other substances in adolescents comprises 6 dichotomous (yes/no) items, with the cut-off point at ≥2 positive items. The internal consistency level of the Spanish psychometric validation was 0.74, the sensitivity was 74.4%, and the specificity was 96.4% [33].

The Substance Use and Abuse Subscale of the Problem-Oriented Screening Instrument for Teenagers (POSIT) assesses the risky use of alcohol and other drugs in adolescents. The scale contains 17 dichotomous (yes/no) items and has a cut-off point of ≥2 positive items; it shows a high value for internal consistency (0.82), sensitivity (94.3%), and specificity (83.9%) [34].

The Video Game-Related Experiences Questionnaire (CERV in Spanish) values the problematic use of non-massive video games. The CERV contains 17 items on worry, denial, increased tolerance, negative effects, reduced activities, loss of control, avoidance, and desire to play and has a cut-off point of ≥26 with a Cronbach alpha of 0.912 [35].

The Game Addiction Scale for Adolescents (GASA) [36] assesses addiction to video games. It consists of 7 items that correspond to a 7-dimensional structure (salience, tolerance, mood modification, relapse, withdrawal, conflict, and problems) that are grouped into a higher order factor: addiction. Each item is assessed dichotomously, and each positive item is summed, with the cut-off point being ≥4. The reliability of the Spanish adaptation was 0.81 [37].

The TXP Parental Socialization Questionnaire, designed for the Spanish population, assesses parental socialization practices. The TXP is subdivided into two questionnaires: TXP-A (applied to adolescents) and TXP-C (applied to the main caregiver). The TXP-A consists of 29 items and provides data on affect-communication and control-structure factors, while the TXP-C comprises 16 items and provides data on affect-communication factors and prosocial values. The TXP shows high internal reliability (0.87) and test-retest reliability 0.94 [38].

The Parental Socialization Styles Scale in Adolescence (ESPA-29) [39] evaluates parental styles and is based on two axes of socialization: implication-acceptance (expression of reactions of approval and affect when children behave in accordance with family norms) and coercion-imposition (a socialization style used when children behave in a manner discrepant with the rules of family functioning). These two dimensions are independent, and four parental styles are obtained from their combination: authoritative, indulgent, authoritarian, and negligent. The ESPA-29 presents high levels of internal consistency between 0.82 and 0.94 [40].

The Big Five Personality Test for Children and Adolescents (BFQ-NA) [41] is an adaptation of the Big Five Personality Model. The internal consistency of the overall scale was 0.86, and by subscales, it was as follows: Consciousness = 0.87, Agreeableness = 0.82, Neuroticism = 0.83, Extraversion = 0.76, and Openness = 0.75 [42].

The Behavior Assessment System for Children (BASC) [43,44] contains 5 components that can be used together or individually. In this current study, we used the Self-Report (S3) questionnaire completed by adolescents and a questionnaire for Parents (P3). The internal consistency of the global dimensions of the BASC was between 0.76 and 0.96, with a mean value of 0.91. S3 provides data from clinical scales and 4 global dimensions: School Maladjustment (SMC), Clinical Maladjustment (CMC), Personal Adjustment (PAC), and the Emotional Symptoms Rate (ESR). The P3 questionnaire measures maladaptive behaviors, which allowed us to obtain values for Externalising problems, Internalising problems, and Adaptive skills, as well as a Behavioural Symptoms Index (BSI).

### 2.3. Procedure

After authorization by the participating educational centers, a letter was sent to the parents/guardians of the students in the third and fourth years of CSE to request authorization for their children to participate in this study. Once the authorization was obtained, the questionnaires were filled out by the students for an hour and a half during school hours on 2 consecutive days. The surveys were completed between October and December 2018 with the supervision of 2 psychologists. The parents/guardians of participating students received the questionnaires by post and returned them to the school. Neither the adolescents nor their relatives received compensation of any type for their collaboration.

With the results obtained in the psychometric tests, three groups were formed: Participants with a high risk of SUD, a strong likelihood of developing SUD (HRSUD; a score above the cut-offs in 2 or 3 of the AUDIT, CRAFFT, and POSIT questionnaires, n = 65), those with a low risk of SUD, these individuals show signs of risk, but their likelihood of developing a SUD is lower compared to the high-risk group (LRSUD; score above the cut-off in only one of the AUDIT, CRAFFT, or POSIT questionnaires, n = 46), and with no addiction, they do not show significant signs of risk for SUD in any of the questionnaires used (NA; scores below the cut-off point in all three questionnaires, n = 220). Participants who presented gaming disorder (score above the cut-off point in the GASA and CERV questionnaires, n = 39) were excluded to avoid possible biases.

### 2.4. Data Analysis

SPSS software (v23, IBM Corp., Armonk, NY, USA) was used to study the relationships between the study variables, using chi-squared (categorical variables) and ANOVA (quantitative variables and categorical variables with more than two categories) tests, considering the results significant when *p* < 0.05. Since multivariate ANOVA is used in each of the multiple comparison tables using F, the reference *p*-value has been included by applying the Bonferroni correction: 0.05/number of variables compared.

To study which independent variables allow HRSUD to be predicted, binary logistic regression models were created: first using the significant variables from the ANOVA and chi-squared tests to obtain the unadjusted odds ratio, and subsequently performing regression modeling using a conditional forward selection method for sociodemographic variables and for each of the TXP, ESPA-29, BFQ-NA, and BASC questionnaires. Finally, a regression model was implemented using a conditional forward selection method adjusted by sociodemographic variables and the TXP, ESPA-29, BFQ-NA, and BASC questionnaires.

## 3. Results

After excluding any adolescents with gaming disorder, the sample size was 356. Of these, 25 participants were eliminated because of missing values, leaving a final study sample of 331 adolescents. Of this sample, 61.8% (n = 220) had NA, 12.9% (n = 46) had a LRSUD, and 18.3% (n = 65) had a HRSUD. Table 1, Table 2, Table 3, Table 4 and Table 5 show the means and proportions for the overall sample, as well as for each of the three groups, and include the significant differences between the three groups in terms of sociodemographic variables, parental socialization, personality, behavior, and psychopathology.

A higher proportion of participants in the fourth year of compulsory secondary education had a LRSUD (17.8%; n = 35) or HRSUD (24.4%; n = 48), and these participants were older (F = 9.16; *p* < 0.001) for LRSUD (M = 15.07; SD = 0.72; *p* = 0.006) and HRSUD (M = 15.06; SD = 0.69; *p* = 0.001) than in the NA group (M = 14.71; SD = 0.70). No differences were found by sex, repeated school years, average grade, type of educational center, family living arrangements, or number of siblings.

Table 6 and Table 7 show the summary of the parental socialization, personality and behavior traits, and psychopathology variables that presented significant differences between the NA, LRSUD, and HRSUD groups. Adolescents with NA scored higher in affect communication than those with a LRSUD (F = 14.15; *p* = 0.003) or HRSUD (F = 14.15; *p* < 0.001). Adolescents with a HRSUD scored higher in maternal indifference (F = 5.02; *p* = 0.043) and paternal indifference (F = 5.86; *p* = 0.008) and lower in control-structure (F = 7.56; *p* = 0.001) than the NA group. No significant differences were found between those with a HRSUD and LRSUD in terms of parenting.

Regarding personality traits, no significant differences were found between those with NA or a LRSUD. The group with NA scored higher in conscientiousness (F = 16.10; *p* < 0.001), openness (F = 8.02; *p* < 0.001), and agreeableness (F = 8.22; *p* = 0.005) and scored lower in neuroticism (F = 21.34; *p* < 0.001) than adolescents with a HRSUD. Adolescents with a LRSUD scored higher in conscientiousness (F = 16.10; *p* = 0.011) and agreeableness (F = 8.22; *p* < 0.001) and scored lower in neuroticism (F = 21.34; *p* < 0.001) than the group with a HRSUD.

Table 8 shows the results of the binary logistic regression with the unadjusted odds ratio (UOR). Table 9 shows the results of the binary logistic regression with the adjusted odds ratio (AOR), which allows us to predict a HRSUD by age (odds ratio [OR] = 2.187; 95% CI [1.118, 4.281]); *p* = 0.022), sensation seeking (OR = 1.084; 95% CI [1.037, 1.133]); *p* < 0.001), and the personality traits of conscientiousness (OR = 0.930; 95% CI [0.882, 0.981]); *p* = 0.008) and neuroticism (OR = 1.049; 95% CI [1.002, 1.098]); *p* = 0.042).

## 4. Discussion

This study had two main objectives. On the one hand, to identify which parental socialization styles influence the development of substance addiction in the adolescent population and, on the other, to determine the influence of personality, excluding participants who presented gaming disorder. In relation to the first objective, our results showed that adolescents who did not meet addiction criteria had higher levels of affect and communication, a finding that has already been demonstrated in previous research, in which affect in family relationships stood out as a protective factor against at the start of substance use [45]. In fact, a worse relationship between parents and children, characterized by less communication and parental control, predisposes to greater alcohol consumption and the initiation of cannabis use [17].

Thus, an upbringing based on communication and affect, typical of the authoritative parental socialization style, is considered a protective factor against substance use, as shown by studies conducted in European and American adolescents [19,46,47,48]. That is, authoritative parenting has been associated with lower levels of alcohol and other substance use among adolescents [46,49,50,51]. Likewise, the establishment and application of clear rules of behavior, or the equivalent of dynamically controlling and supervising the activities of children, are factors that prevent substance use [13,48,49,52,53]. In addition, maternal control has been shown to be a more important protective factor against substance use [54].

On the other hand, the negligent and authoritarian parenting styles pose a greater risk of substance abuse in adolescents [11,12,13], while the permissive style, despite not being beneficial, did not imply additional risk in this context [45]. These findings are in line with those from our study, which found that adolescents with a high risk of substance use disorder had higher scores on maternal indifference and paternal indifference and lower scores on parental control, corroborating that youngsters who grow up in families in which parents were less involved were less effective in refusing substances and with it, had a higher frequency of consumption [55].

In terms of our objective of evaluating the relationship between personality traits and substance use, we found that non-addicted adolescents with a LRSUD presented higher scores in conscientiousness, extroversion, and agreeableness and lower scores in neuroticism compared to adolescents with a HRSUD. Similar results were shown in previous research with the five-factor personality model, with most work indicating a significant association between neuroticism, low agreeableness and conscientiousness, and problematic alcohol use [23,56,57]. In the same way, other studies have concluded that, regardless of the types of substances consumed, the most common personality traits are high neuroticism and low conscientiousness [23,58].

In the regression analysis, the variables of age, neuroticism, and sensation seeking were found to be risk factors for substance abuse, while conscientiousness was a protective factor. Similar findings were shown in the study by Tsavou & Petkari (2020), where neuroticism was postulated as a risk factor in substance abuse [59]. The fact that less control and structure in the family environment is related to a greater risk of substance use could be explained because less parental control and worse family dynamics could predict higher levels of sensation seeking and greater negative emotional symptoms [60,61]. That is, as shown by Rodríguez Rodríguez et al. (2005) when faced with negative emotional symptoms, adolescents would resort to substance use as a sensation-seeking strategy to cope with their emotional difficulties [62]. Thus, Martin et al. (2002) reported that people with a high degree of sensation-seeking showed a high consumption of nicotine, alcohol, and marijuana, presumably in an increased search for sensations, leading to higher levels of substance consumption [63,64,65]. In this same line, Chen et al. (2019) concluded that sensation seeking may be the most important personality trait that differentiates illicit drug users from people with other substance use problems [66]. Thus, it appears to play a role in early, ongoing, and increasing substance use [67].

Consequently, these parental prevention programs can improve communication and positive interactions, thus favoring better family dynamics. Moreover, it seems that the intensity of the program also has a direct impact on its effectiveness, so family intervention with repeated sessions in schools over several weeks provided better results in terms of substance use prevention [68,69].

Regarding age, some studies have described a lower positive influence of the affectionate family environment among older adolescents [70,71], which would also support our study results showing a higher prevalence of older individuals among those at risk for SUD. It should be noted that no differences were found in any of the other sociodemographic variables we measured (sex, repeated school years, average school grade, type of educational center, family living arrangements, or number of siblings). However, contradictory findings were obtained in some previous studies, with results pointing to higher scores in substance use among male adolescents [59,70] and others finding no differences based on sex [71]. Given that parental socialization, in addition to having a direct effect on consumption and addiction, can also influence the personality of adolescents, interventions for the prevention of substance use based on parental practices are necessary. In fact, there is evidence that family intervention can be a preventive method for reducing substance use [72,73].

Regarding the limitations of this work, it should be noted that we decided to exclude participants who presented with gaming disorder but ignored the presence of other behavioral addictions. In addition, the cross-sectional nature of the study did not allow us to infer causality, only relationships. Furthermore, the measures used were self-reported, which can lead to biases such as social desirability. It must also be taken into account that multiple variables have been compared in the three groups studied, which increases the probability of type I error. Given the high number of questionnaires with which adolescents are evaluated, biases may have occurred due to fatigue. So many instruments have been used to minimize the possibility of false positives. Still, more variables that may influence the results could have been evaluated: the social and financial status, the emotional atmosphere in the family (for example, whether there has been a recent bereavement), employment during the day (additional sections, amount of free time), etc. Another possible source of bias was that the sample selection we employed was not random. Regarding the methodology, regression models have been chosen to model the data. Although these models take into account the shared explanation of the variance between the variables studied, other models, such as PROCESS or structural equations, could have taken more account of the interaction. Finally, the strengths of the study were that we neutralized the possible bias of gaming disorder, used two parental socialization scales to obtain more information about this factor; we assessed both adolescents and their caregivers, allowing for a broader perspective, and included the same study variables that are usually studied separately (parental socialization, personality, psychopathology, and substance use).

## 5. Conclusions

Substance use in adolescents could be related to parental socialization (low affect and communication, low control and structure, high maternal indifference, and high paternal indifference) and personality traits (low conscientiousness, low extraversion, low agreeableness, and high neuroticism). Risk factors for substance addiction in adolescents were older age, high neuroticism, and high sensation seeking, while high conscientiousness is a protection factor.

## Figures and Tables

**Table 1:** Sociodemographic characteristics of the adolescents included in this study (n = 331).

	NA	LRSUD	HRSUD	Statistics
Female Gender	63.2% (n = 129)	16.2% (n = 33)	20.6% (n = 42)	χ^2^ 3.07 (*p* = 0.216)
Male Gender	71.7% (n = 91)	10.2% (n = 13)	18.1% (n = 23)	χ^2^ 3.07 (*p* = 0.216)
Age in years	M (SD) = 14.71 (0.70)	M (SD) = 15.07 (0.72)	M (SD) = 15.06 (0.69)	**F 9.16 (*p* < 0.001)** **Tukey’s HSD (*p*)** **LRSUD > NA 0.006** **HRSUD > NA 0.001**
Third year of compulsory secondary education	79.1% (n = 106)	8.2% (n = 11)	12.7% (n = 17)	**χ^2^ 16.19 (*p* < 0.001)** **CTR NA 4.0** **CTR LRSUD** **-2.5** **CTR HRSUD** **-2.6**
Fourth year of compulsory secondary education	57.9% (n = 114)	17.8% (n = 35)	24.4% (n = 48)	χ^2^ 16.19 (*p* < 0.001) CTR NA-4.0 CTR LRSUD 2.5 CTR HRSUD 2.6
No repeated courses	68.5% (n = 161)	13.6% (n = 32)	17.9% (n = 42)	χ^2^ 7.61 (*p* = 0.107)
1 repeated course	47.2% (n = 17)	19.4% (n = 7)	33.3% (n = 12)	χ^2^ 7.61 (*p* = 0.107)
2 repeated courses	55.6% (n = 10)	22.2% (n = 4)	22.2% (n = 4)	χ^2^ 7.61 (*p* = 0.107)
Average grade: Fail	30.8% (n = 4)	30.8% (n = 4)	38.5% (n = 5)	χ^2^ 9.91 (*p* = 0.271)
Average grade: Pass	66.7% (n = 24)	8.3% (n = 3)	25% (n = 9)	χ^2^ 9.91 (*p* = 0.271)
Average grade: Satisfactory	65.7% (n = 46)	15.7% (n = 11)	18.6% (n = 13)	χ^2^ 9.91 (*p* = 0.271)
Average grade: Remarkable	66.7% (n = 78)	14.5% (n = 17)	18.8% (n = 22)	χ^2^ 9.91 (*p* = 0.271)
Average grade: Outstanding	72.9% (n = 35)	12.5% (n = 6)	14.6% (n = 7)	χ^2^ 9.91 (*p* = 0.271)
Secular center	70.3% (n = 147)	13.9% (n = 29)	15.8% (n = 33)	χ^2^ 5.55 (*p* = 0.620)
Catholic center	59.8% (n = 73)	13.9% (n = 17)	26.2% (n = 32)	χ^2^ 5.55 (*p* = 0.620)
Private center	71.2% (n = 79)	15.3% (n = 17)	13.5% (n = 15)	χ^2^ 4.88 (*p* = 0.300)
Chartered (state-subsidized) center	60.6% (n = 57)	14.9% (n = 14)	24.5% (n = 23)	χ^2^ 4.88 (*p* = 0.300)
Public center	66.7% (n = 84)	11.9% (n = 15)	21.4% (n = 27)	χ^2^ 4.88 (*p* = 0.300)
Number of siblings	M (SD) = 2.05 (0.99)	M (SD) = 2.23 (1.01)	M (SD) = 2.17 (0.75)	F 0.82 (*p* = 0.443)
Living with both parents	64.9% (n = 146)	16% (n = 36)	19.1% (n = 43)	χ^2^ 2.10 (*p* = 0.717)
Living with one parent alone	67.2% (n = 41)	9.8% (n = 6)	23% (n = 14)	χ^2^ 2.10 (*p* = 0.717)
Living with other cohabitants	50% (n = 2)	25% (n = 1)	25% (n = 1)	χ^2^ 2.10 (*p* = 0.717)

CTR—corrected typified residuals; F—ANOVA statistic; HRSUD—high risk of substance use disorder; HSD—Honestly-significant-difference; LRSUD—low risk of substance use disorder; M—mean; n—number of participants; NA—no addiction; *p*—*p* value; SD—standard deviation; χ^2^—chi-squared; significant results are shown in bold.

**Table 2:** Personality traits of the adolescents included in this study (n = 331).

	NA M (SD)	LRSUD M (SD)	HRSUD M (SD)	Statistics F (*p*) Tukey’s HSD (*p*)
Conscientiousness (BFQ-NA)	56.34 (9.21)	54.20 (8.79)	49.14 (8.40)	**F 16.10 (*p* < 0.001)** **NA > LRSUD < 0.001** **LRSUD > HRSUD 0.011**
Openness (BFQ-NA)	57.71 (9.50)	56.28 (8.26)	52.51 (8.81)	**F 8.02 (*p* < 0.001)** **NA > HRSUD < 0.001**
Extraversion (BFQ-NA)	50.53 (9.71)	52.93 (9.25)	49.98 (10.21)	F 1.41 (*p* = 0.246)
Agreeableness (BFQ-NA)	53.89 (9.20)	56.61 (8.91)	49.80 (8.99)	**F 8.22 (*p* < 0.001)** **NA > HRSUD 0.005** **LRSUD > HRSUD < 0.001**
Neuroticism (BFQ-NA)	47.46 (10.82)	49.0 (10.76)	57.45 (10.98)	**F 21.34 (*p* < 0.001)** **HRSUD > NA < 0.001** **HRSUD > LRSUD < 0.001**

BFQ-NA—Big Five Questionnaire—Children and Adolescents; F—ANOVA statistic; HRSUD—high risk of substance use disorder; HSD—Honestly-significant-difference; LRSUD—low risk of substance use disorder; M—mean; n—number of participants; NA—no addiction; *p*—*p* value; SD—standard deviation; significant results are shown in bold. Bonferroni adjustment: *p* = 0.01.

**Table 3:** Behavior (BASC-S3) of the adolescents included in this study (n = 331).

	NA M (SD)	LRSUD M (SD)	HRSUD M (SD)	Statistics F (*p*) Tukey’s HSD (*p*)
Negative attitude to school (BASC-S3)	48.08 (9.65)	53.33 (11.44)	54.95 (11.61)	**F 13.60 (*p* < 0.001)** **LRSUD > NA 0.014** **HRSUD > NA < 0.001**
Negative attitude to teachers (BASC-S3)	45.58 (9.27)	47.98 (10.17)	51.37 (10.44)	**F 9.28 (*p* < 0.001)** **HRSUD > NA < 0.001**
Sensation seeking (BASC-S3)	46.74 (9.92)	49.83 (10.47)	54.60 (9.72)	**F 15.94 (*p* < 0.001)** **HRSUD > NA < 0.001** **HRSUD > LRSUD 0.035**
Atypicality (BASC-S3)	46.04 (8.19)	47.87 (10.67)	54.25 (9.63)	**F 21.45 (*p* < 0.001)** **HRSUD > NA < 0.001** **HRSUD > LRSUD 0.005**
Locus of control (BASC-S3)	45.04 (9.02)	49.80 (11.96)	53.37 (13.04)	**F 17.66 (*p* < 0.001)** **LRSUD > NA 0.035** **HRSUD > NA < 0.001**
Somatization (BASC-S3)	49.00 (9.47)	51.46 (11.48)	55.57 (12.80)	**F 9.99 (*p* < 0.001)** **HRSUD > NA 0.001**
Social stress (BASC-S3)	47.88 (9.26)	52.00 (12.93)	53.06 (11.16)	**F 8.06 (*p* < 0.001)** **HRSUD > NA 0.003**
Anxiety (BASC-S3)	48.24 (10.23)	51.02 (10.59)	53.09 (9.94)	**F 6.16 (*p* = 0.002)** **HRSUD > NA 0.003**
Depression (BASC-S3)	47.88 (9.27)	52.07 (11.20)	54.06 (14.18)	**F 9.72 (*p* < 0.001)** **HRSUD > NA 0.004**
Sense of inadequacy (BASC-S3)	49.07 (9.38)	50.91 (10.60)	54.60 (12.92)	**F 7.23 (*p* = 0.001)** **HRSUD > NA 0.005**
Interpersonal relations (BASC-S3)	50.34 (9.40)	48.76 (11.85)	48.35 (10.01)	F 1.26 (*p* = 0.284)
Relations with parents (BASC-S3)	52.13 (9.48)	50.07 (11.16)	44.17 (15.43)	**F 12.82 (*p* < 0.001)** **NA > HRSUD 0.001**
Self-esteem (BASC-S3)	51.70 (8.24)	48.26 (10.66)	48.03 (11.44)	**F 5.44 (*p* = 0.005)** **NA > HRSUD 0.047**
Self-confidence (BASC-S3)	49.70 (8.73)	48.74 (10.08)	44.34 (10.60)	**F 8.29 (*p* < 0.001)** **NA > HRSUD < 0.001** **LRSUD > HRSUD 0.039**
Clinical maladjustment (BASC-S3)	46.41 (9.11)	50.24 (11.73)	55.65 (11.38)	**F 21.93 (*p* < 0.001)** **HRSUD > NA < 0.001** **HRSUD > LRSUD 0.045**
School maladjustment (BASC-S3)	46.06 (9.19)	50.50 (10.34)	54.98 (10.70)	**F 22.56 (*p* < 0.001)** **LRSUD > NA 0.014** **HRSUD > NA < 0.001** **HRSUD > LRSUD 0.044**
Personal adjustment (BASC-S3)	51.22 (8.95)	48.22 (10.87)	44.69 (12.13)	**F 11.23 (*p* < 0.001)** **NA > HRSUD < 0.001**
ESR (BASC-S3)	48.19 (9.36)	52.07 (11.64)	54.00 (11.33)	**F 9.48 (*p* < 0.001)** **HRSUD > NA 0.001**

BASC-S3—Behavior Assessment System for Children Self-Report; ESR—Emotional Symptoms Rate; F—ANOVA statistic; HRSUD—high risk of substance use disorder; HSD—Honestly-significant-difference; LRSUD—low risk of substance use disorder; M—mean; n—number of participants; NA—no addiction; *p*—*p* value; SD—standard deviation; significant results are shown in bold. Bonferroni adjustment: *p* = 0.002.

**Table 4:** Behavior (BASC-P3) of the adolescents included in this study (n = 331).

	NA M (SD)	LRSUD M (SD)	HRSUD M (SD)	Statistics F (*p*) Tukey’s HSD (*p*)
Aggression (BASC-P3)	43.23 (7.61)	44.92 (8.89)	46.31 (9.00)	**F 3.21 (*p* = 0.042)** **HRSUD > NA 0.042**
Hyperactivity (BASC-P3)	43.84 (8.33)	47.83 (10.01)	46.26 (10.02)	**F 3.88 (*p* = 0.022)** **LRSUD > NA 0.037**
Conduct Problems (BASC-P3)	45.08 (8.10)	49.39 (10.58)	51.84 (12.86)	**F 11.58 (*p* < 0.001)** **HRSUD > NA 0.002**
Attention Problems (BASC-P3)	46.18 (9.58)	47.83 (9.32)	48.96 (10.06)	F 1.84 (*p* = 0.161)
Atypicality (BASC-P3)	47.03 (9.59)	50.14 (12.45)	49.50 (13.30)	F 1.97 (*p* = 0.142)
Depression (BASC-P3)	44.45 (8.08)	48.44 (14.33)	46.98 (9.81)	**F 3.55 (*p* = 0.030)**
Anxiety (BASC-P3)	46.71 (9.07)	48.78 (10.56)	50.61 (11.10)	**F 3.54 (*p* = 0.030)**
Withdrawal (BASC-P3)	50.75 (9.67)	50.17 (11.67)	49.40 (11.03)	F 0.36 (*p* = 0.697)
Somatization (BASC-P3)	46.83 (7.86)	49.67 (12.38)	49.44 (13.25)	F 2.32 (*p* = 0.100)
Social skills (BASC-P3)	54.50 (9.69)	53.17 (8.37)	52.12 (10.39)	F 1.32 (*p* = 0.270)
Leadership (BASC-P3)	52.85 (9.91)	54.03 (9.70)	53.88 (11.68)	F 0.31 (*p* = 0.711)
Externalising problems (BASC-P3)	43.89 (7.98)	47.75 (10.40)	49.14 (11.09)	**F 8.39 (*p* < 0.001)** **HRSUD > NA 0.007**
Internalising problems (BASC-P3)	45.22 (8.07)	49.08 (13.83)	49.04 (12.79)	**F 3.42 (*p* = 0.013)**
Adaptability (BASC-P3)	54.24 (10.09)	54.19 (8.69)	53.34 (11.41)	F 0.16 (*p* = 0.855)
BSI (BASC-P3)	43.90 (9.13)	47.78 (12.19)	48.08 (11.77)	**F 4.80 (*p* = 0.009)**

BASC-P3—Behavior Assessment System for Children for Parents; BSI—Behavioural Symptoms Index; F—ANOVA statistic; HRSUD—high risk of substance use disorder; HSD—Honestly-significant-difference; LRSUD—low risk of substance use disorder; M—mean; n—number of participants; NA—no addiction; *p*—*p* value; SD—standard deviation; significant results are shown in bold. Bonferroni adjustment: *p* = 0.003.

**Table 5:** Parental socialization of the adolescents included in this study (n = 331).

	NA M (SD)	LRSUD M (SD)	HRSUD M (SD)	Statistics F (*p*) Tukey’s HSD (*p*)
Affect-Communication (TXP-A)	86.34 (13.01)	78.40 (14.13)	77.08 (16.70)	**F 14.15 (*p* < 0.001)** **NA > LRSUD 0.003** **NA > HRSUD < 0.001**
Control-Structure (TXP-A)	35.76 (5.68)	33.55 (6.24)	32.83 (5.96)	**F 7.56 (*p* = 0.001)** **HRSUD > NA < 0.001**
Prosocial Values (TXP-C)	19.44 (1.53)	19.19 (1.43)	19.27 (1.44)	F 0.56 (*p* = 0.570)
Affect-Communication (TXP-C)	55.56 (7.66)	53.32 (7.23)	53.84 (6.91)	F 1.68 (*p* = 0.188)
Mother’s Reasoning (ESPA-29)	3.00 (0.68)	2.88 (0.76)	2.90 (0.78)	F 0.60 (*p* = 0.551)
Mother’s Warmth (ESPA-29)	3.03 (0.79)	2.82 (0.79)	2.89 (0.82)	F 1.40 (*p* = 0.249)
Mother’s Detachment (ESPA-29)	1.28 (0.30)	1.34 (0.36)	1.47 (0.50)	**F 5.02 (*p* = 0.007)** **HRSUD > NA 0.043**
Mother’s Indifference (ESPA-29)	1.70 (0.73)	1.89 (0.80)	1.85 (0.73)	F 1.40 (*p* = 0.248)
Mother’s Physical Punishment (ESPA-29)	1.04 (0.13)	1.07 (0.15)	1.08 (0.14)	F 1.65 (*p* = 0.193)
Mother’s Revoking Privileges (ESPA-29)	1.67 (0.64)	1.57 (0.54)	1.72 (0.59)	F 0.63 (*p* = 0.533)
Mother’s Verbal Scolding (ESPA-29)	2.54 (0.71)	2.41 (0.63)	2.63 (0.62)	F 0.94 (*p* = 0.394)
Mother’s Acceptance/Involvement (ESPA-29)	3.27 (0.48)	3.15 (0.50)	3.14 (0.56)	F 1.39 (*p* = 0.252)
Mother’s Strictness/Imposition (ESPA-29)	1.76 (0.43)	1.72 (0.39)	1.81 (0.35)	F 0.52 (*p* = 0.593)
Father’s Reasoning (ESPA-29)	2.82 (0.77)	2.59 (0.82)	2.65 (0.71)	F 2.01 (*p* = 0.137)
Father’s Warmth (ESPA-29)	2.86 (0.84)	2.53 (0.91)	2.65 (0.86)	F 3.01 (*p* = 0.051)
Father’s Detachment (ESPA-29)	1.39 (0.51)	1.52 (0.52)	1.59 (0.45)	**F 3.20 (*p* = 0.042)**
Father’s Indifference (ESPA-29)	1.82 (0.78)	2.15 (0.93)	2.21 (0.78)	**F 5.86 (*p* = 0.003)** **HRSUD > NA 0.008**
Father’s Physical Punishment (ESPA-29)	1.03 (0.13)	1.03 (0.09)	1.09 (0.22)	**F 3.68 (*p* = 0.026)**
Father’s Revoking Privileges (ESPA-29)	1.60 (0.63)	1.48 (0.50)	1.62 (0.51)	F 0.74 (*p* = 0.480)
Father’s Verbal Scolding (ESPA-29)	2.36 (0.67)	2.24 (0.63)	2.40 (0.58)	F 0.73 (*p* = 0.484)
Father’s Acceptance/Involvement (ESPA-29)	3.10 (0.58)	2.89 (0.59)	2.91 (0.55)	F 2.93 (*p* = 0.055)
Father’s Strictness/Imposition (ESPA-29)	1.66 (0.39)	1.58 (0.32)	1.70 (0.33)	F 1.31 (*p* = 0.272)

ESPA-29—Parental Socialization Styles Scale in Adolescence; F—ANOVA statistic; HRSUD—high risk of substance use disorder; HSD—Honestly-significant-difference; LRSUD—low risk of substance use disorder; M—mean; n—number of participants; NA—no addiction; *p*—*p* value; SD—standard deviation; TXP-A—TXP Parental Socialization Questionnaire applied to adolescents; TXP-C—TXP Parental Socialization Questionnaire applied to the main caregiver; significant results are shown in bold. Bonferroni adjustment: *p* = 0.002.

**Table 6:** Differences between individuals with no addiction and a high or low risk of substance use disorder (ANOVA: F[*p*]).

	NA		
	Parental Socialization	Personality Traits	Behaviour and Psychopathology
LRSUD	Affect-Communication (TXP-A) 14.15 (0.003) ^NA^		Negative attitude to school 13.60 (0.014) ^NA^ Locus of control 17.66 (0.035) ^LRSUD^ School maladjustment 22.56 (0.014) ^LRSUD^ Hyperactivity 3.88 (0.037) ^LRSUD^
HRSUD	Affect-Communication (TXP-A) 14.15 (<0.001) ^NA^ Control and adolescent structure 7.56 (0.001) ^NA^ Mother’s Detachment 5.02 (0.043) ^HRSUD^ Father’s Indifference 5.86 (0.008) ^HRSUD^	Conscientiousness 16.10 (<0.001) ^NA^ Openness 8.02 (<0.001) ^NA^ Agreeableness 8.22 (0.005) ^NA^ Neuroticism 21.34 (<0.001) ^HRSUD^	Negative attitude to school 13.60 (<0.001) ^NA^ Negative attitude to teachers 9.28 (<0.001) ^NA^ Sensation seeking 15.94 (<0.001) ^HRSUD^ Atypicality (BASC-S3) 21.45 (<0.001) ^HRSUD^ Locus of control 17.66 (<0.001) ^HRSUD^ Somatization 9.99 (0.001) ^HRSUD^ Social stress 8.06 (0.003) ^HRSUD^ Anxiety (BASC-S3) 6.16 (0.003) ^HRSUD^ Depression (BASC-S3) 9.72 (0.004) ^HRSUD^ Sense of inadequacy 7.23 (0.005) ^HRSUD^ Relations with parents 12.82 (0.001) ^NA^ Self-esteem 5.44 (0.047) ^NA^ Self-confidence 8.29 (<0.001) ^NA^ Clinical maladjustment 21.93 (<0.001) ^HRSUD^ School maladjustment 22.56 (<0.001) ^HRSUD^ Personal adjustment 11.23 (<0.001) ^NA^ ESR 9.48 (0.001) ^HRSUD^ Aggression 3.21 (0.042) ^HRSUD^ Conduct problems 11.58 (0.002) ^HRSUD^ Externalising problems 8.39 (0.007) ^HRSUD^

BASC-S3—Behavior Assessment System for Children Self-Report; ESR—Emotional Symptoms Rate; F—ANOVA statistic; HRSUD—high risk of substance use disorder; LRSUD—low risk of substance use disorder; NA—no addiction; *p*—*p* value; TXP-A—TXP Parental Socialization Questionnaire applied to adolescents; The name of the group (NA, LRSUD or HRSUD) that scored highest in Tukey post hoc tests for homogeneous variance or in Games–Howell post hoc significance comparison tests for non-homogeneous variance (*p* < 0.05) is shown after each variable in superscript.

**Table 7:** Differences between individuals with a high or low risk of substance use disorder (ANOVA: F[*p*]).

	**LRSUD**	
	**Personality Traits**	**Behaviour and Psychopathology**
HRSUD	Conscientiousness 16.10 (0.011) ^LRSUD^ Agreeableness 8.22 (<0.001) ^LRSUD^ Neuroticism 21.34 (<0.001) ^HRSUD^	Sensation seeking 15.94 (0.035) ^HRSUD^ Atypicality (BASC-S3) 21.45 (0.005) ^HRSUD^ Self-confidence 8.29 (0.039) ^LRSUD^ Clinical maladjustment 21.93 (0.045) ^HRSUD^ School maladjustment 22.56 (0.044) ^HRSUD^

BASC-S3—Behavior Assessment System for Children Self-Report; F—ANOVA statistic; HRSUD—high risk of substance use disorder; LRSUD—low risk of substance use disorder; *p*—*p* value; The name of the group (LRSUD or HRSUD) that scored highest in Tukey post hoc tests for homogeneous variance or in Games–Howell post hoc significance comparison tests for non-homogeneous variance (*p* < 0.05) are shown after each variable in superscript.

**Table 8:** The unadjusted odds ratio of the binary logistic regression used to predict High Risk of Substance Use Disorder.

Independent Variables	UOR [95% CI], *p*
Age	1.981 [1.331–2.948], 0.001
Fourth compulsory secondary education year	2.625 [1.422–4.847], 0.002
Affect-Communication (TXP-A)	0.962 [0.944–0.979], <0.001
Control-Structure (TXP-A)	0.922 [0.880–0.967], 0.001
Mother’s Detachment	3.501 [1.502–8.159], 0.004
Father’s Indifference	1.783 [1.216–2.616], 0.003
Conscientiousness	0.915 [0.883–0.947], <0.001
Openness	0.943 [0.915–0.973], <0.001
Agreeableness	0.952 [0.923–0.983], 0.002
Neuroticism	1.087 [1.056–1.118], <0.001
Relations with parents	0.950 [0.929–0.972], <0.001
Self-esteem	0.962 [0.936–0.989], 0.006
Self-confidence	0.945 [0.918–0.972], <0.001
Personal adjustment	0.944 [0.919–0.970], <0.001
Negative attitude to school	1.060 [1.033–1.088], <0.001
Negative attitude to teachers	1.059 [1.030–1.088], <0.001
Sensation seeking	1.078 [1.047–1.110], <0.001
Atypicality (BASC-S3)	1.098 [1.063–1.133], <0.001
Locus of control	1.072 [1.044–1.101], <0.001
Somatization	1.053 [1.028–1.080], <0.001
Social stress	1.049 [1.021–1.077], <0.001
Anxiety (BASC-S3)	1.050 [1.020–1.082], 0.001
Depression (BASC-S3)	1.046 [1.022–1.071], <0.001
Sense of inadequacy	1.048 [1.022–1.075], <0.001
Clinical maladjustment	1.090 [1.058–1.123], <0.001
School maladjustment	1.092 [1.059–1.126], <0.001
Emotional symptoms rate	1.053 [1.026–1.081], <0.001
Aggression	1.046 [1.008–1.084], 0.017
Conduct problems	1.068 [1.034–1.104], <0.001
Externalising problems	1.062 [1.027–1.099], 0.001

95% CI—95% confidence interval; BASC-S3—Behavior Assessment System for Children Self-Report; *p*—*p* value; TXP-A—TXP Parental Socialization Questionnaire applied to adolescents; UOR—Unadjusted Odds Ratio.

**Table 9:** The odds ratio adjusted by age, parenting, personality, behavior, and psychopathology of a binary logistic regression model by using a conditional forward selection method to predict High Risk of Substance Use Disorder.

Independent Variables	AOR [95% CI], *p*
Age	2.187 [1.118–4.281], 0.022
Conscientiousness	0.930 [0.882–0.981], 0.008
Neuroticism	1.049 [1.002–1.098], 0.042
Sensation seeking	1.084 [1.037–1.133], <0.001

95% CI—95% confidence interval; AOR—Adjusted Odds Ratio; *p*—*p* value.

## Data Availability

The data presented in this study are available on request from the corresponding author. The data are not publicly available due to privacy restrictions.
